# Development of a new scoring system for predicting recurrent thrombosis in patients with antiphospholipid syndrome

**DOI:** 10.1186/s42358-025-00476-1

**Published:** 2025-09-24

**Authors:** Oh Chan Kwon, Jang Woo Ha, Min-Chan Park, Yong-Beom Park, Sang-Won Lee

**Affiliations:** 1 Division of Rheumatology, Department of Internal Medicine, Gangnam Severance Hospital, Yonsei University College of Medicine, Seoul, Korea; 2 Division of Rheumatology, Department of Internal Medicine, Yongin Severance Hospital, Yonsei University College of Medicine, Seoul, Korea; 3 Division of Rheumatology, Department of Internal Medicine, Severance Hospital, Yonsei University College of Medicine, 50-1 Yonsei-ro, Seodaemun-gu, Seoul, 03722 Republic of Korea

**Keywords:** Antiphospholipid syndrome, Thrombosis, Risk assessment

## Abstract

**Background:**

This study aimed to develop a new scoring system for predicting recurrent thrombosis in patients with antiphospholipid syndrome (APS).

**Methods:**

This was a retrospective multicentre cohort study. Patients with APS were followed up from APS diagnosis to either recurrent thrombosis or their last follow-up date, whichever came first. A multivariable logistic regression analysis with a backward elimination was used to develop a new scoring system, incorporating conventional cardiovascular risk factors and antiphospholipid antibody (aPL) profile as covariates. Each covariate remaining in the final step was multiplied by its β coefficient, rounded to the nearest integer, and then summed. A multivariable Cox proportional hazard model, adjusted for medication use, assessed the association between the new scoring system and recurrent thrombosis risk. The accuracy of the new scoring system in predicting recurrent thrombosis was evaluated by estimating the area under the curve (AUC) and comparing it with the adjusted global APS score (aGAPSS).

**Results:**

A total of 126 patients with APS were included. The median age of the cohort was 40 (interquartile range: 31–55) years and 49.2% were women. Hypertension, type 2 diabetes mellitus (T2DM), and dyslipidaemia were present in 20.6%, 18.3%, and 54.0% of the patients, respectively. 26.2% were current smokers and 23.8% had concomitant systemic lupus erythematosus. Lupus anticoagulant (LA), anti-β2 glycoprotein I (anti-β2GPI) IgG, anti-β2GPI IgM, anti-cardiolipin (aCL) IgG, and aCL IgM were positive in 76.2%, 27.0%, 11.9%, 27.0%, and 6.3% of the patients, respectively. During a median follow-up duration of 41.5 (interquartile range: 21.4–72.0) months, thrombosis recurred in 9 (7.1%) patients. Multivariable logistic regression analysis revealed the following scoring system: modified diabetes global APS score (mdGAPSS) = 1*T2DM + 2*LA + 1*anti-β2GPI. Multivariable Cox proportional hazard model showed that a higher score was significantly associated with an increased risk of recurrent thrombosis (adjusted hazard ratio 2.911, 95% confidence interval 1.090–7.770, *p* = 0.033). The AUC was higher using the mdGAPSS (AUC = 0.706) than the aGAPSS (AUC = 0.553).

**Conclusions:**

Compared to the aGAPSS, the mdGAPSS—comprising T2DM, LA, and anti-β2GPI—demonstrated a greater accuracy in predicting recurrent thrombosis in patients with APS.

## Background

Antiphospholipid syndrome (APS) is characterized by recurrent venous or arterial thrombosis and/or pregnancy complications in the presence of persistent antiphospholipid autoantibodies (aPL) [1]. Despite the use of anticoagulants, many patients continue to experience recurring thrombosis. A prospective observational study spanning 10 years reported a recurrent thrombosis rate of 16.6% in the first 5 years and 14.4% in the subsequent 5 years among patients with APS [2]. Importantly, thrombotic event is the most common cause of death in patients with APS [2]. Hence, it is crucial to stratify the risk of recurrent thrombosis in patients with APS to ensure stringent monitoring of those deemed high-risk.

For the risk prediction of thrombosis, the global APS score (GAPSS)—a risk score that incorporates conventional cardiovascular risk factors and aPL—has been developed [3]. The GAPSS was initially developed in patients with systemic lupus erythematosus [3] and subsequently validated in patients with primary APS [4]. The GAPSS consists of hyperlipidaemia, arterial hypertension, anti-cardiolipin (aCL) immunoglobulin (Ig) G/IgM, anti-β2 glycoprotein I (anti-β2GPI) IgG/IgM, anti-phosphatidylserine/prothrombin (anti-PS/PT) IgG/IgM, and lupus anticoagulant (LA) [3]. As anti-aPS/PT IgG/IgM are not routinely tested in most clinical laboratories, an alternative risk score, the adjusted GAPSS (aGAPSS), which excludes anti-PS/PT from the GAPSS, has also been developed and validated [3, 5, 6]. Notably, although type 2 diabetes mellitus (T2DM) is considered an important conventional cardiovascular risk factor [7, 8], it is not included in the GAPSS or aGAPSS. In the development cohort of GAPSS, only two of 106 patients (1.9%) had T2DM [3], and the low prevalence of T2DM in that cohort could have resulted in an underestimation of the impact of T2DM on thrombosis risk. Previous studies have reported a high prevalence of T2DM in patients with APS, ranging from 7.0 to 8.7% [9, 10]. Given that T2DM is one of the major risk factors for cardiovascular disease [7, 8], we speculate that the scoring system could be more accurate on incorporating T2DM. In this study, we therefore aimed to develop a new scoring system that includes T2DM for predicting recurrent thrombosis in patients with APS using a multicentre cohort.

## Methods

### Study cohort

This was a retrospective multicentre cohort study. Patients diagnosed with APS at two referral hospitals were retrospectively included for analysis. All patients fulfilled the 2006 revised Sapporo classification criteria for APS [11]. The observation period was from the date of APS diagnosis to the date of recurrent thrombosis or the last date of follow-up, whichever came first. The following covariates at the time of APS diagnosis were collected: age, sex, presence of hypertension, T2DM, and dyslipidaemia, smoking status (current smoker or not), concomitant systemic lupus erythematosus (SLE), index clinical event of APS (arterial thrombosis, venous thrombosis, both arterial and venous thrombosis, stroke, acute coronary syndrome, and pulmonary thromboembolism), aPL profile (LA, anti-β2GPI IgG/IgM, and aCL IgG/IgM), and aGAPSS. As anti-PS/PT were not tested in the cohort patients, we used aGAPSS instead of GAPSS. LA was tested using an ACL TOP 700 coagulation analyser (Instrumentation Laboratory, Milan, Italy) with an assay kit utilising diluted Russell’s viper venom (HemosIL Diluted Russell’s Viper Venom Time Screen/Confirm kit, Instrumentation Laboratory). The aCL IgG/IgM and anti-β2GPI IgG/IgM were measured using an automated fluorescence enzyme immunoassay (EliA; Phadia, Sweden). Medium-to-high titres (> 40 units) of aCL IgG/IgM and anti-β2 GPI IgG/IgM were considered positive. Data on the use of antiplatelets, anticoagulants (warfarin, low molecular weight heparin, and direct oral anticoagulants), statins, glucocorticoids, hydroxychloroquine, and immunosuppressants during observation period were also collected. Patients with conventional cardiovascular risk factors (hypertension, T2DM, and dyslipidaemia) were managed for their respective conditions using antihypertensive agents, antidiabetic medications, and statins, respectively.

This study was approved by the Institutional Review Board (IRB) of Severance Hospital (IRB No: 4-2024-0628) and was conducted in accordance with the Declaration of Helsinki. The requirement for informed consent was waived owing to the retrospective nature of the study.

### Definition of recurrent thrombosis

Recurrent thrombosis was defined as an unequivocal arterial or venous thrombosis detected in imaging studies performed for the evaluation of symptomatic events. The remaining thrombosis from the index event of APS was not considered a recurrence.

### Statistical analysis

Descriptive analysis was used for summarizing patients’ characteristics. Continuous variables are expressed as median (interquartile range) and categorical variables as number (%). To develop a new scoring system for predicting recurrent thrombosis, we conducted a multivariable logistic regression analysis with a backward elimination method. Conventional cardiovascular risk factors and aPL profile were included as covariates in the multivariable model, and covariates with *p* > 0.2 were eliminated in each step. The use of antihypertensive agents, antidiabetic medications, and statins was not included in the multivariable model because the use of each medication is closely related to its corresponding condition (i.e., hypertension, T2DM, and dyslipidaemia), introducing multicollinearity into the model. Therefore, we chose to include the underlying risk factors themselves rather than their treatment proxies to ensure model stability. We derived a novel equation for the prediction of recurrent thrombosis using the β coefficients of variables remaining in the final step of the multivariable logistic regression analysis. The β coefficients were rounded to the nearest integer (simplified β). The weights of the rounded β coefficients were assigned to each variable according to the slopes like a coefficient of a linear equation. Next, a multivariable Cox proportional hazard regression model adjusted for the use of antiplatelets, anticoagulants, statins, and glucocorticoids was used to assess the association between the new scoring system and the risk of recurrent thrombosis. To test the accuracy of the new scoring system in predicting recurrent thrombosis, we analysed the receiver operating characteristic (ROC) curve and estimated the area under the curve (AUC). For comparison, the AUC of aGAPSS in our cohort was also assessed. Statistical significance was set at *p* < 0.05. All analyses were conducted using SPSS software version 26.0 (IBM Corporation, Armonk, NY, USA).

## Results

### Characteristics of the cohort

A total of 126 patients with APS were included; their characteristics are summarized in Table 1. The median age of the cohort was 40 (interquartile range: 31–55) years and 49.2% were women. Regarding the conventional cardiovascular risk factors, hypertension, T2DM, and dyslipidaemia were identified in 20.6%, 18.3%, and 54.0% of the patients, respectively, and 26.2% were current smokers. Thirty (23.8%) of the patients had concomitant SLE. In terms of aPL profile, LA, anti-β2GPI IgG, anti-β2GPI IgM, aCL IgG, and aCL IgM were positive in 76.2%, 27.0%, 11.9%, 27.0%, and 6.3% of the patients, respectively. The median value of aGAPSS was 7 (interquartile range: 4–11). During a median follow-up duration of 41.5 (interquartile range: 21.4–72.0) months, thrombosis recurred in 9 (7.1%) patients.

**Table 1:** Characteristics of 126 patients with APS

		Values		
*At the time of diagnosis*				
Demographic data				
Age (years)		40 (31–55)		
Female sex (N (%))		62 (49.2)		
Hypertension		26/118 (20.6)		
T2DM		23/115 (18.3)		
Dyslipidaemia		68 (54.0)		
Smoking		33/117 (26.2)		
Concomitant SLE		30 (23.8)		
Clinical items				
Thrombosis		115 (91.3)		
Arterial thrombus		72 (57.1)		
Venous thrombus		56 (44.4)		
Both Artery and venous		13 (10.3)		
Stroke		41 (32.5)		
Acute coronary syndrome		8 (6.3)		
Pulmonary thromboembolism		33 (26.2)		
Gestational problem		16 (12.7)		
1 ≥ Foetal death at or beyond the 10th week of gestation		6 (4.8)		
3 ≥ Consecutive abortion before the 10th week of gestation		5 (4.0)		
1 ≥ Premature birth before the 34th week of gestation because of eclampsia, severe preeclampsia, or placental insufficiency		5 (4.0)		
Immunologic items				
LA		96 (76.2)		
Anti-β2GPI IgG		34 (27.0)		
Anti-β2GPI IgM		15 (11.9)		
aCL IgG		34 (27.0)		
aCL IgM		8 (6.3)		
aGAPSS		7 (4–11)		
*Medications during follow-up*				
Antiplatelets		64 (50.8)		
Anticoagulants				
None		28 (22.2)		
Warfarin		65 (51.6)		
LMWH		5 (4.0)		
DOAC		28 (22.2)		
Statins		68 (54.0)		
Cumulative dose* of glucocorticoids (mg)		0.0 (0.0–1575.0)		
Hydroxychloroquine		38 (30.2)		
Immunosuppressants				
Azathioprine		7 (5.6)		
Cyclophosphamide		1 (0.8)		
Cyclosporin A		2 (1.6)		
Methotrexate		4 (3.2)		
Mycophenolate mofetil		9 (7.1)		
Rituximab		1 (0.8)		
Tacrolimus		10 (7.9)		
*Follow-up*				
Follow-up duration (month)		41.5 (21.4–72.0)		
Recurrence of thrombus		9 (7.1)		

Values are expressed as median (interquartile range) or number (percentage)

APS: Antiphospholipid syndrome; SLE: systemic lupus erythematosus; T2DM: type 2 diabetes mellitus; LA: lupus anticoagulant; Anti-β2GPI: anti- β2 glycoprotein I; Ig: immunoglobulin; aCL: anti-cardiolipin; aGAPSS: adjusted global antiphospholipid syndrome score; LMWH: low molecular weight heparin; DOAC: direct oral anticoagulant

*Mg of prednisolone or its equivalent

### New scoring system development

The results of the multivariable logistic regression analysis are presented in Table 2. T2DM, LA, and anti-β2GPI were retained in the final step. Specifically, the β coefficients of T2DM, LA, and anti-β2GPI were 1.348, 1.565, and 1.224, respectively. Using simplified β coefficients of each variable, we developed a new scoring system as follows: modified diabetes GAPSS (mdGAPSS) = 1*T2DM (presence = 1 or absence = 0) + 2*LA (presence = 1 or absence = 0) + 1*anti-β2GPI (presence = 1 or absence = 0).

**Table 2:** Multivariable logistic regression analysis for the development of a new scoring system

	β coefficient	Simplified β
Diabetes	1.348	1
LA	1.565	2
Anti-β2GPI	1.224	1

LA: lupus anticoagulant; Anti-β2GPI: anti- β2 glycoprotein I

Modified diabetes global antiphospholipid syndrome score = 1*type 2 diabetes mellitus (presence = 1 or absence = 0) + 2*LA (presence = 1 or absence = 0) + 1*anti-β2GPI (presence = 1 or absence = 0)

### Association between the MdGAPSS and risk of recurrent thrombosis

We employed Cox proportional hazard models to examine whether the mdGAPSS is associated with recurrent thrombosis risk. In the univariable model, higher mdGAPSS was significantly associated with an increased risk of recurrent thrombosis (unadjusted hazard ratio [HR] 2.328, 95% confidence interval [CI] 1.035–5.239, *p* = 0.041). Furthermore, even after adjusting for the use of antiplatelets, anticoagulants, statins, and glucocorticoids in the multivariable model, a higher mdGAPSS remained significantly associated with a heightened risk of recurrent thrombosis (adjusted HR 2.911, 95% CI 1.090–7.770, *p* = 0.033) (Table 3).

**Table 3:** Association between the MdGAPSS and risk of recurrent thrombosis

	Univariable model		Multivariable model	
	Unadjusted HR (95% CI)	P value	Adjusted HR (95% CI)	P value
mdGAPSS	2.328 (1.035–5.239)	0.041	2.911 (1.090–7.770)	0.033
Antiplatelets	1.426 (0.355–5.728)	0.617	3.439 (0.689–17.152)	0.132
Anticoagulants				
None	1.000 (ref)		1.000 (ref)	
Warfarin	2.127 (0.255–17.749)	0.486	2.496 (0.252–24.696)	0.434
LMWH	0.000 (N/A)	0.989	0.000 (N/A)	0.989
DOAC	1.942 (0.120–31.477)	0.641	2.154 (0.119–38.984)	0.603
Statins	0.525 (0.140–1.966)	0.338	0.375 (0.088–1.600)	0.185
Glucocorticoids	1.000 (1.000–1.000)	0.406	1.000 (1.000–1.000)	0.209

mdGAPSS: modified diabetes global antiphospholipid syndrome score; HR: hazard ratio; CI: confidence interval; LMWH: low molecular weight heparin; DOAC: direct oral anticoagulant

### Accuracy of the MdGAPSS in predicting recurrent thrombosis

**Fig. 1:**
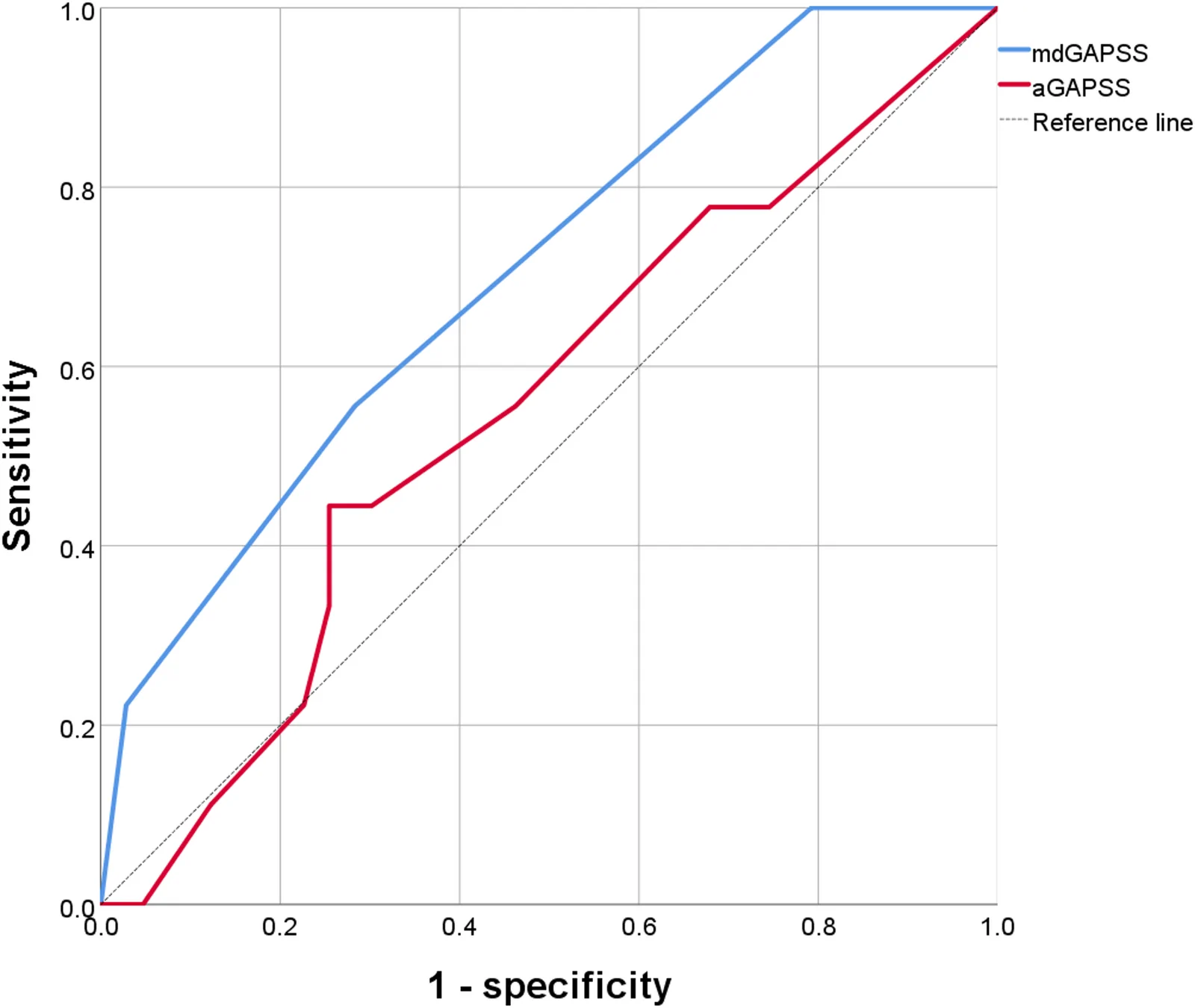
ROC curve analysis using the mdGAPSS and aGAPSS as test variables. ROC: receiver operating characteristic; mdGAPSS: modified diabetes global antiphospholipid syndrome; aGAPSS: adjusted global antiphospholipid syndrome score

The ROC curves of the mdGAPSS and aGAPSS are shown in Fig. 1. The AUC of the mdGAPSS was 0.706 (95% CI 0.536–0.876, *p* = 0.041), which was higher than that of aGAPSS (AUC 0.553, 95% CI 0.361–0.745, *p* = 0.593).

## Discussion

In this study, we developed a new scoring system, the mdGAPSS, that could predict recurrent thrombosis in patients with APS. The mdGAPSS consisted of T2DM (1 point), LA (2 points), and anti-β2GPI (1 point). A higher score was significantly associated with a higher risk of recurrent thrombosis. Notably, the mdGAPSS had a higher accuracy in predicting recurrent thrombosis than the aGAPSS. Considering the critical need for accurate risk stratification of thrombosis in patients with APS [12], the newly developed scoring system in our study is of clinical importance.

One of the main differences between mdGAPSS and the aGAPSS is the inclusion of T2DM rather than arterial hypertension and dyslipidaemia. This difference could be attributable to the different characteristics of the patients included in our cohort and that in the development cohort of GAPSS [3]. In our cohort, the prevalence of T2DM was 18.3%, which is higher than that in the development cohort of GAPSS (1.9%) [3]. With a higher prevalence of T2DM in the cohort, we were able to assess T2DM more rigorously as a potential covariate to be included in the scoring system. In the multivariable logistic regression analysis, T2DM but not arterial hypertension and dyslipidaemia remained in the final step. This suggests that among the conventional cardiovascular risk factors, T2DM could be more important than arterial hypertension or dyslipidaemia in stratifying thrombotic risk. T2DM is the most potent conventional cardiovascular risk factor, which is considered a cardiovascular disease risk equivalent (i.e., risk of developing cardiovascular disease 20% over 10 years) [13, 14]. Our finding indicates that T2DM should be considered an important factor when assessing recurrent thrombotic risk in patients with APS.

Another major difference between mdGAPSS and the aGAPSS is that the weight of the aPL profile is different. In the aGAPSS, LA, anti-β2GPI, and aCL were weighted 4 points, 4 points, and 5 points, respectively [4]. That is, aCL was weighted as the highest score among the aPLs. In contrast, in our study, LA was weighted 2 points, anti-β2GPI was weighted 1 point, and aCL was not included in the scoring system. Studies have reported that the odds ratio for thrombosis is highest with LA, followed by anti-β2GPI and aCL [15, 16]. Given this different risk according to the aPL profile, the higher weight of LA than anti-β2GPI and the exclusion of aCL in the mdGAPSS seem more plausible than the aGAPSS. These differences could have led to a more accurate prediction of recurrent thrombosis using the mdGAPSS than the aGAPSS.

Previous studies did not consider the effect of medications when assessing the association between GAPSS or aGAPSS and the risk of thrombosis [3–6, 17, 18]. In our study, however, we adjusted for the use of antiplatelets, anticoagulants, statins, and glucocorticoids in the multivariable Cox proportional hazard regression model. As the number of events was relatively small, we were unable to adjust for all medications, including hydroxychloroquine and immunosuppressants, due to an overfitting issue. We specifically chose to adjust for antiplatelets, anticoagulants, statins, and glucocorticoids as covariates because these medications are closely related to thrombosis risk [19–21]. An advantage of our study is the adjustment of these medications, which was not performed in previous studies. We found that the higher score of the mdGAPSS was significantly associated with a higher risk of recurrent thrombosis, even after adjusting for the use of these medications.

Another point to note is that the prevalence of dyslipidaemia is high in patients with APS. In our cohort, the prevalence of dyslipidaemia was 54.0%, which is similar to the previous studies that reported a dyslipidaemia prevalence of 35.1–53.2% [4, 22]. Despite the high prevalence, dyslipidaemia often remains undertreated [22]. Given that statins exert potent antithrombotic effects in addition to lipid-lowering effects, particularly in the context of arterial thrombosis [23, 24], their use merits particular attention.

This study has some limitations. Firstly, the number of events observed was insufficient to draw strong conclusions. Further studies with a larger sample size would be helpful in validating our new scoring system. Secondly, our study exclusively enrolled Korean participants. Given the varying prevalence of conventional cardiovascular risk factors across different ethnic populations [25], it is essential to conduct validation studies in other ethnic populations. Lastly, this study employed a retrospective design. Therefore, the potential influence of unmeasured confounders, such as compliance to medications and time in therapeutic range for warfarin, should be considered when interpreting our data. In addition, regarding diabetes and its metabolic control, HbA1c data were not consistently available across the cohort, limiting our ability to assess glycaemic control as a covariate.

## Conclusions

In conclusion, we developed a new scoring system, the mdGAPSS, which consists of T2DM, LA, and anti-β2GPI (1*T2DM [presence = 1 or absence = 0] + 2*LA [presence = 1 or absence = 0] + 1*anti-β2GPI [presence = 1 or absence = 0]). The mdGAPSS had a higher accuracy in predicting recurrent thrombosis in patients with APS than the aGAPSS. This new scoring system is easy to calculate and has the potential to enhance clinicians’ ability to accurately predict the risk of recurrent thrombosis.

## Data Availability

All data generated or analysed during this study are included in this article.

## Abbreviations

APS: Antiphospholipid syndrome
aPL: antiphospholipid autoantibodies
GAPSS: Global antiphospholipid syndrome score
aCL: anti-cardiolipin
Ig: Immunoglobulin
anti-β2GPI: anti-β2 glycoprotein I
anti-PS/PT: anti-phosphatidylserine/prothrombin
LA: Lupus anticoagulant
aGAPSS: adjusted global antiphospholipid syndrome score
T2DM: Type 2 diabetes mellitus
IRB: Institutional Review Board
ROC: Receiver operating characteristic
AUC: Area under the curve
mdGAPSS: modified diabetes global antiphospholipid syndrome score
HR: Hazard ratio
CI: Confidence interval
